# Age-Specific Epigenetic Drift in Late-Onset Alzheimer's Disease

**DOI:** 10.1371/journal.pone.0002698

**Published:** 2008-07-16

**Authors:** Sun-Chong Wang, Beatrice Oelze, Axel Schumacher

**Affiliations:** 1 Epigenetics Lab, Department of Medicine II, Klinikum rechts der Isar, Munich, Germany; 2 Institute of Systems Biology and Bioinformatics, National Central University, Jhongli City, Taiwan; 3 Sequenom GmbH, Hamburg, Germany; Ohio State University Medical Center, United States of America

## Abstract

Despite an enormous research effort, most cases of late-onset Alzheimer's disease (LOAD) still remain unexplained and the current biomedical science is still a long way from the ultimate goal of revealing clear risk factors that can help in the diagnosis, prevention and treatment of the disease. Current theories about the development of LOAD hinge on the premise that Alzheimer's arises mainly from heritable causes. Yet, the complex, non-Mendelian disease etiology suggests that an epigenetic component could be involved. Using MALDI-TOF mass spectrometry in post-mortem brain samples and lymphocytes, we have performed an analysis of DNA methylation across 12 potential Alzheimer's susceptibility loci. In the LOAD brain samples we identified a notably age-specific epigenetic drift, supporting a potential role of epigenetic effects in the development of the disease. Additionally, we found that some genes that participate in amyloid-β processing (PSEN1, APOE) and methylation homeostasis (MTHFR, DNMT1) show a significant interindividual epigenetic variability, which may contribute to LOAD predisposition. The APOE gene was found to be of bimodal structure, with a hypomethylated CpG-poor promoter and a fully methylated 3′-CpG-island, that contains the sequences for the ε4-haplotype, which is the only undisputed genetic risk factor for LOAD. Aberrant epigenetic control in this CpG-island may contribute to LOAD pathology. We propose that epigenetic drift is likely to be a substantial mechanism predisposing individuals to LOAD and contributing to the course of disease.

## Introduction

Alzheimer's disease (AD) is the most prominent form of dementia among the elderly. Despite enormous research efforts, the etiology of AD remains obscure and puzzling. Although some genes for some early-onset familial forms of Alzheimer's disease have been identified (∼5% of cases), the overwhelming proportion of diagnosed AD cases remains unexplained. These circumstances led to a rethinking of the classical molecular approaches, shifting the emphasis from genetic causative factors to epigenetic and environmental effects. Yet, empirical support for specific environmental risk has been very inconsistent [1]. Genetic influences seem to play a more significant role in the onset of the rare early-onset form of AD (EOAD) [2]. In these cases mutations in the amyloid-β precursor protein (APP), and the presenilin genes PSEN1 and PSEN2 are known to be associated with autosomal dominant EOAD. A different picture seems to emerge for late-onset AD (LOAD), a common sporadic form of the illness affecting individuals above the age of 65 years. In contrast to monogenic diseases, LOAD exhibits numerous non-Mendelian anomalies that suggest an epigenetic component in disease etiology. Such anomalies include among others: 1.) Sporadic cases dominate over familial ones; 2.) estimated concordance rates for monozygotic twins are significantly below 100%, a hallmark of complex non-Mendelian diseases; 3.) differential susceptibility and course of illness in males and females [3], [4]; 4.) parent-of-origin effects [5]; 5.) late age of onset; 6.) brain chromatin abnormalities, including aberrant histone modifications; 7.) non-Mendelian inheritance pattern; 8.) abnormal levels of folate and homocysteine, indicative of an abnormal methylation homeostasis in the brain of AD patients; 9.) a disturbed control of the epigenetically regulated circadian clock and 10.) monoallelic expression patterns of susceptibility genes [6]. Compared to genetic causes, epigenetic factors are probably much more suited to explain the observed anomalies in LOAD as aberrant epigenetic patterns may be acquired during many developmental stages. The epigenome is particularly susceptible to deregulation during early embryonal and neonatal development, puberty and especially old age [7], which is the most important known risk factor for AD.

Surprisingly, little is known about the role of an epigenetic component in the development of AD. One study from the early 90's on one post-mortem brain sample of an unaffected patient suggested that the APP promoter is always unmethylated in brain and hence may not be controlled by DNA methylation in the brain of healthy individuals [8]. However, this study did not compare statistical amounts of samples; neither did the study reveal whether DNA methylation of the interrogated sites is present in AD patients. Another study by Schwob *et al.* found no significant difference in total percent methylation of CCGG sites from brain DNA of AD patients compared with 20 normal subjects [9]. However, this method did not possess the sensitivity to distinguish between different DNA methylation profiles, genomic distribution of methylcytosine nor if potentially disturbed methylation patterns exist in a subpopulation of cells. It is now acknowledged that it is important to know more about the epigenetic patterns of the genes involved in AD pathogenesis to understand the mechanisms that regulate gene function and to potentially enable pharmacological intervention on the epigenetic level.

In this study we asked whether DNA methylation patterns in post-mortem brains and lymphocytes from LOAD patients are different from patterns found in healthy individuals and if age affects the distribution of these profiles. Hence, we performed a hypothesis-driven analysis of DNA methylation patterns across candidate genes for which *a priori* evidence for a role in the etiology of AD exists. Additionally, we analysed the promoter methylation of genes which are essential components of the epigenetic machinery and thus may serve as indicators of global epigenetic malfunctions in LOAD. Here, we demonstrate that LOAD patients have a larger epigenetic distance from the norm in brain tissue compared with controls and that the epigenetic distance increases with age, supporting a role of epigenetic effects in the development of the disease. Some genes that play central roles in amyloid-β processing (i.e. PSEN1 and APOE) and methylation homeostasis (i.e. MTHFR and DNMT1) also show a significant interindividual epigenetic variability, which may contribute to AD predisposition.

## Results

DNA Methylation analysis was performed using base-specific cleavage of single-stranded nucleic acids with MALDI-TOF mass spectrometry analysis of the cleavage products [10], [11] (for details see supplementary Material & Methods S1). All measurements were highly reproducible and 123 of the 124 CpG units analyzed in this study yielded successful measurements in >98% of samples. Of the total 5796 interrogated CpG units in the 46 individuals, only 59 units (∼1%) could not be analyzed.

### Overall DNA methylation

**Figure 1:**
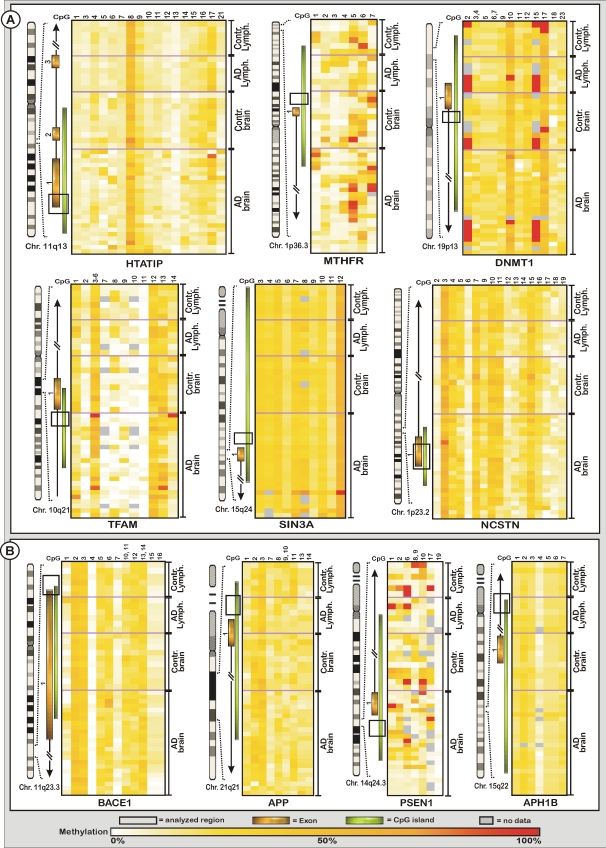
DNA methylation profiles of GC-rich regions in potential AD-susceptibility genes. A: Genes involved in LOAD or genes that are a central part of the epigenetic machinery of the cell. A strong interindividual variance in DNA methylation could be observed within the promoters of MTHFR, DNMT1 for cases and controls and in TFAM for LOAD patients. Note that all ‘abnormal’ patterns within HTATIP, NCSTN, TFAM or SIN3A are from LOAD cases and exclusively observed in brain tissue. B: Methylation profiles of the promoter region of the APP gene and genes involved in APP processing. PSEN1 demonstrated the highest interindividual variation of all genes analyzed, whereas BACE, APP and APH1B did not display any significant variation in the analyzed individuals.

From the 12 analyzed CpG-rich regions, 9 were mostly unmethylated (<20% methylation), 2 gene promoters were partly methylated (20–50%) and only one hypermethylated (>50% methylation). This result is in agreement with earlier observation of CpG island methylation patterns on chromosome 6, 20 and 22, where the majority of CpG islands (CGIs) were unmethylated and only a small fraction (9.2%) were hypermethylated [12]. The hypermethylated CGI was the only region analyzed outside of a promoter region. Such hypermethylated loci are generally associated with a closed chromatin structure [13], whereas the analyzed 5′-promoter regions might reflect an open chromatin structure. Most genes such as APP, NCSTN, BACE, SIN3A, APH1B, HTATIP or DNMT1 revealed the same methylation patterns in the majority of brain tissues and in the lymphocytes (Fig. 1). Although some interesting age- and gene-specific trends could be observed, no significant changes in overall methylation patterns could be observed in AD patients compared to the controls (data not shown).

Neither brain samples nor lymphocytes exhibited significant overall methylation difference between the sexes. Similarly, no significant tissue-specific differences in methylation levels could be observed. Generally, it is assumed that tissue-specific transcription is controlled by tissue-specific differentially methylated regions (T-DMRs). Such regulatory elements are essential for specifying tissue type identity in mammals. Hierarchical clustering of our data showed that biological replicates of both tissue types did not cluster together (data not shown), indicating a lack of tissue-specific methylation profiles in the analyzed genes. This result is not surprising, since none of the analyzed genes are believed to have a role in tissue differentiation mechanisms and because only a small fraction of the loci in the human genome are differentially methylated in different tissues [12], [14]. Furthermore, according to online expression databases, most of the analyzed genes are abundantly expressed in multiple tissues including brain and blood. Only 5 genes (APOE, TFAM, APP, APH1B and DNMT1) demonstrate major expression differences in the studied tissues; however, the influence of DNA methylation on their promoter is poorly studied. For APP, the relationship between promoter methylation and gene expression has been explored. It was found that the promoter region in humans and primates displayed tissue- and brain-region-specific profiles of methylation, which crudely reflect APP expression patterns [15]. Similarly, a direct relationship between methylation level and promoter activity are known for PSEN1 and BACE [16]. Both genes are expressed at high levels in brain cells as well as in lymphocytes and are, as expected, unmethylated in both tissues.

**Figure 2:**
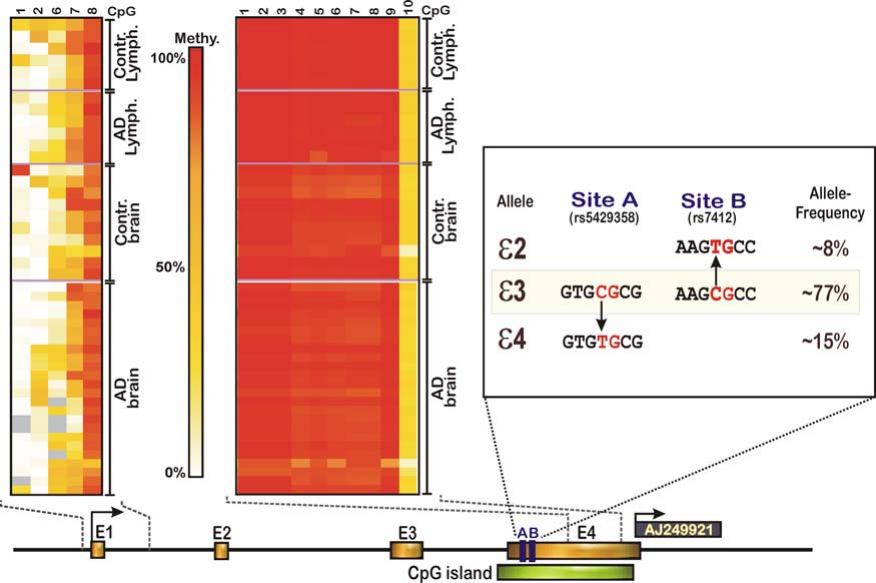
DNA methylation and epigenetic variance in the APOE gene. A: DNA methylation increases from the upstream core promoter gradually towards intron 1. The first exon is non-coding, exon 2 codes for the signal peptide and exons 3 and 4 for the mature protein. The internal 3′-CGI, covering exon 4, displays hypermethylation in all individuals. The 3′-CGI contains the sequences for the ε2, ε3 and ε4 haplotypes. The ε4 haplotype of APOE is the only undisputed genetic risk factor for late-onset Alzheimer's disease. Interindividual variance of DNA methylation was notably in the APOE promoter but not in the 3′-CGI. B: The occurrence of an ε2 or ε4 haplotype removes a CpG dinucleotides from the 3′-CpG island potentially affecting the higher order chromatin structure, that could result in aberrant regulation of APOE and the downstream transcript AJ249921. C = Controls, AD = Alzheimer patients; Ly = Lymphocytes, Br = brain.

Of all the genes analysed, only within the gene for apolipoprotein E (APOE) a hypermethylated CGI could be identified (see Fig. 2a). Interestingly, the APOE gene belongs to a group of genes that do not possess a classical CpG island in their promoters, but rather a low-CpG density region [17]. However, the gene contains a high-density CGI at its 3′end that covers exon 4, which contains the sequences for the major haplotypes (*ε*2–*ε*4), which determine risk to develop AD. APOE is the prime candidate for late-onset Alzheimer's disease and patients are routinely screened for these APOE genotypes. It is not known if the internal CGI possesses a regulatory function, however brain specific transcripts originating directly downstream of this CGI (i.e. AJ249921) were previously identified. DNA methylation patterns within the 3′-CGI were close to 100% in all individuals, with the exception of one LOAD patient who had a methylation of below 80% averaged over the 10 CpG sites analysed. In total, the interindividual variation within the 3′-CGI was very low (see Fig. 2b). The opposite pattern could be observed for the GC-poor 5′-promoter, which was hypomethylated in all individuals, however with a much higher degree of interindividual variance. The methylation increased downstream of the core promoter, which spans from about 500 bp 5′ to the transcription start site up to 300 bp in intron 1. The impact of DNA methylation on APOE promoter activity itself is unknown since the regulation of APOE is highly complex and does not only rely on the 5′-promoter, it also requires an interaction of proximal and distal regulatory regions with transcription factors to impart a net effect on APOE expression. In human brain, most of the *cis*-acting variance in APOE expression is accounted for by the *ε*4 haplotype, but there are additional, small, *cis*-acting influences associated with promoter genotypes [18]. The *ε*4 sequence (that is usually associated with a higher risk of LOAD) may change the epigenetic function of the methylated 3′-CGI since the *ε*4 allele introduces a C → T transition that is associated with a loss of a methylatable CpG unit (Fig. 2c). The risk associated with the *ε*4 allele is dose dependent and it was shown previously that the relative APOE *ε*4 mRNA level is increased in AD compared to controls, suggesting that variability in the neuronal expression of APOE contributes to disease risk [19], [20].

### Epigenetic distance

**Figure 3:**
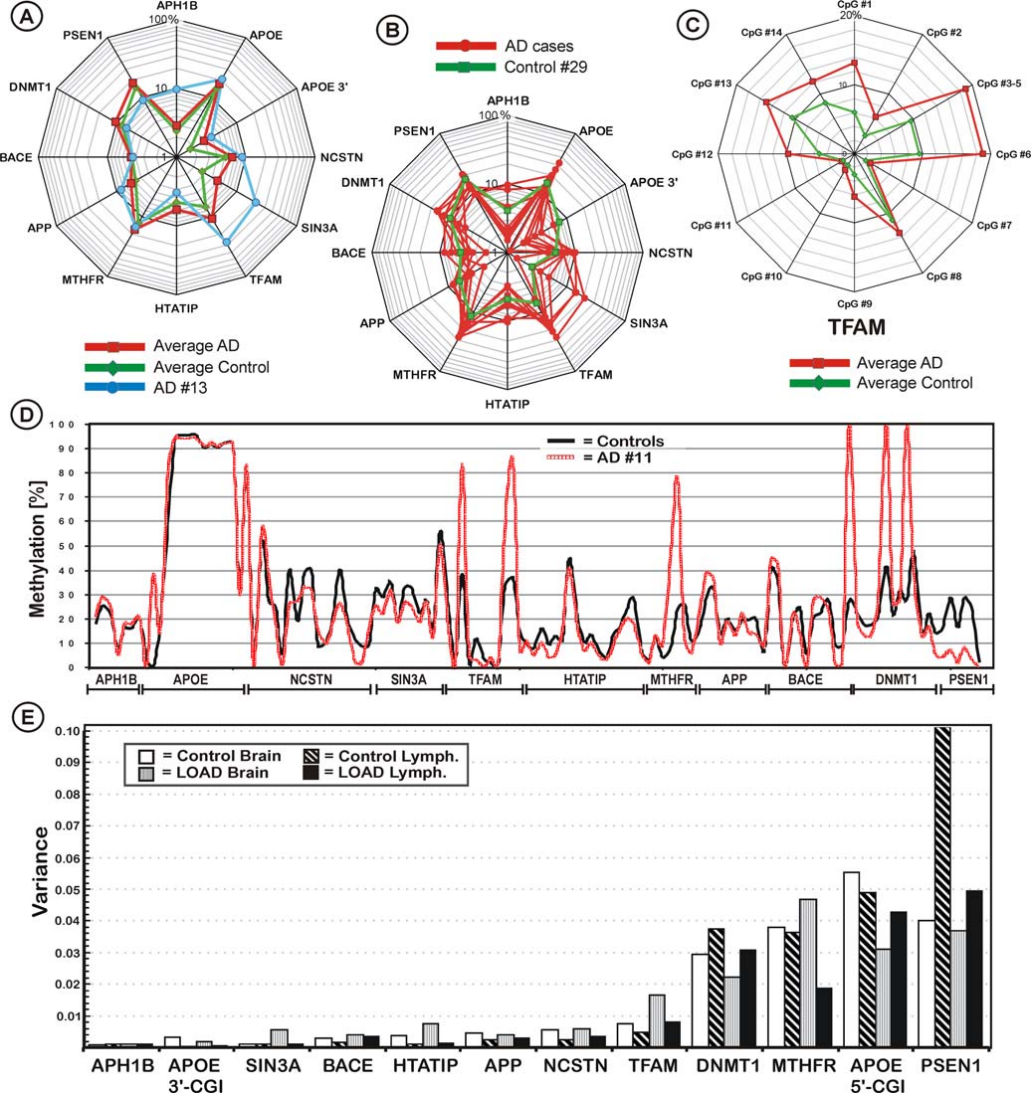
DNA methylation difference from the norm in brain. A: The AD cases show the largest epigenetic distance, measured as the average absolute methylation difference from the norm in all genes. B: The 10 individuals with the biggest epigenetic difference to the norm. Only one control individual (#29) displayed a notably epigenetic distance from the norm, whereas all other individuals with notable epigenetic distances are LOAD cases. C: DNA methylation differences in the TFAM gene between AD cases and controls. D: Example of an AD case (#11) with large epigenetic distance to the norm. The brain DNA of this 94 year old female shows a hypomethylation of PSEN1, whereas the APOE promoter, DNMT1, MTHFR and TFAM are hypermethylated compared to the norm. E: Interindividual variation of DNA methylation levels in brain and lymphocytes of LOAD patients and controls. The largest interindividual variance was observed for the promoters of PSEN1 and APOE, which are the prime candidates for LOAD susceptibility.

To test if there is a significant epigenetic difference between the healthy control group and LOAD patients and to characterize individuals depending on their epigenetic profile, we analyzed the epigenetic distance of each individual to the norm (the median methylation of the healthy control individuals). The epigenetic distance was represented either as the Euclidean distance of the profile of an individual compared to the group of unaffected controls (see Materials and Methods) or as absolute methylation difference between an individual and the norm at a site (Fig. 3), since any epigenetic deregulation can involve demethylation as well as de novo methylation. Interestingly, for all analysed genes we found that the methylation patterns in brains of LOAD cases were slightly more dissimilar to the norm compared with control brains (Fig. 3a). Some individuals, as exemplified with LOAD patient #13, displayed a significant epigenetic distance. In total, 9 out of the 10 most ‘abnormal’ methylation patterns were observed in LOAD patients and only one in a control brain sample (control #29; Fig. 3b). Of all the genes, the gene for mitochondrial transcription factor A (TFAM), a key activator of mitochondrial transcription in mammals, displayed the strongest epigenetic distance in LOAD brains. In total, 16 out of 24 LOAD patients (67%) displayed a notable epigenetic distance from the norm in TFAM, whereas only one of the controls did.

Figure 3d exemplifies a typical methylation pattern of one LOAD brain sample from a 94 year old female patient (AD #11), that displays a relative large epigenetic distance from the norm in the putative LOAD susceptibility genes PSEN1, APOE and TFAM as well as in DNMT1 and MTHFR. Similar patterns were observed in several individuals, reflected in the fact that the genes deregulated in the aged brain of individual #11 are the most variant genes in all individuals. In general, most genes displayed a very low interindividual variance in all analysed individuals, however, four CpG islands displayed a moderate to large interindividual variance, especially the promoters of DNMT1, MTHFR, APOE and largest in PSEN1 (Fig. 3e). The most variable single CpG sites were observed for DNMT1 CpG's #2 and #15 and MTHFR CpG's #5 and #7. No significant tissue- or gender-specific variance could be identified.

### Age dependent epigenetic drift

**Figure 4:**
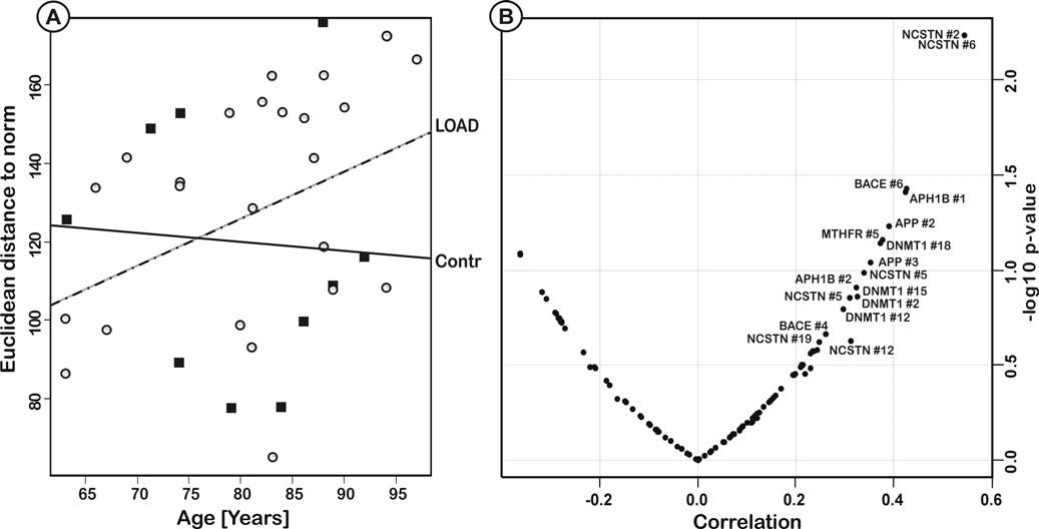
Age-dependent epigenetic drift in brain samples. A: The epigenetic Euclidean distance of LOAD patients compared to the ‘epigenetic norm’ increases with age (Pearson product-moment correlation coefficient = 0.41, p = 0.045), whereas no significant drift could be identified for the control individuals. Circles = LOAD cases, Boxes = controls. B: Correlation between epigenetic drift and age of single CpG sites in LOAD post-mortem brain samples (Spearman correlation). Several of the sites display a significant age-dependent drift in the LOAD patients; most notably in the NCSTN and DNMT1 promoter sequences.

By calculating the Euclidean distance to the norm for each methylation profile, we found a notable epigenetic drift from the norm in the brains of the LOAD patients but not in controls (see Fig. 4a). According to the theory of epigenetic drift (see discussion), AD patients may undergo an enhanced epigenetic drift or alternatively their epigenomes were already at an advanced level of abnormality earlier in life, for example due to the influence of environmental factors, transgenerational effects or by disruption of the epigenetic machinery. A sign of potential deregulation of the epigenomic machinery in the brains of affected individuals may be the observation that aberrant DNA methylation patterns are not uniform and occur either as demethylation or as de novo methylation. Indeed, for the majority of the analyzed CpG islands, we could not observe a directed methylation change although the Euclidean distance increased at the same time. Some of the genes displayed a bivalent distribution of methylcytosine with age, characterized by a trend towards a progressive demethylation with age in normal control brains, whereas the LOAD brains displayed the opposite pattern with an increase in DNA methylation in these CGIs. The opposite pattern was strongest for MTHFR and APOE. This result is especially interesting for the APOE promoter, since APOE is the main candidate for LOAD. On average, the methylation level of MTHFR and APOE in individuals 30 years of age apart decreased in the control brains by 10.6%, whereas the AD patients methylation level increased by 6.8% (see Supporting Fig. S2). Although some of the genes behaved similar in lymphocytes, the overall trend was different and could not be reproduced in lymphocytes. Similarly, no gender-specific methylation changes with age could be identified (see Supporting Table S2).

### Epigenetic disease signatures

**Figure 5:**
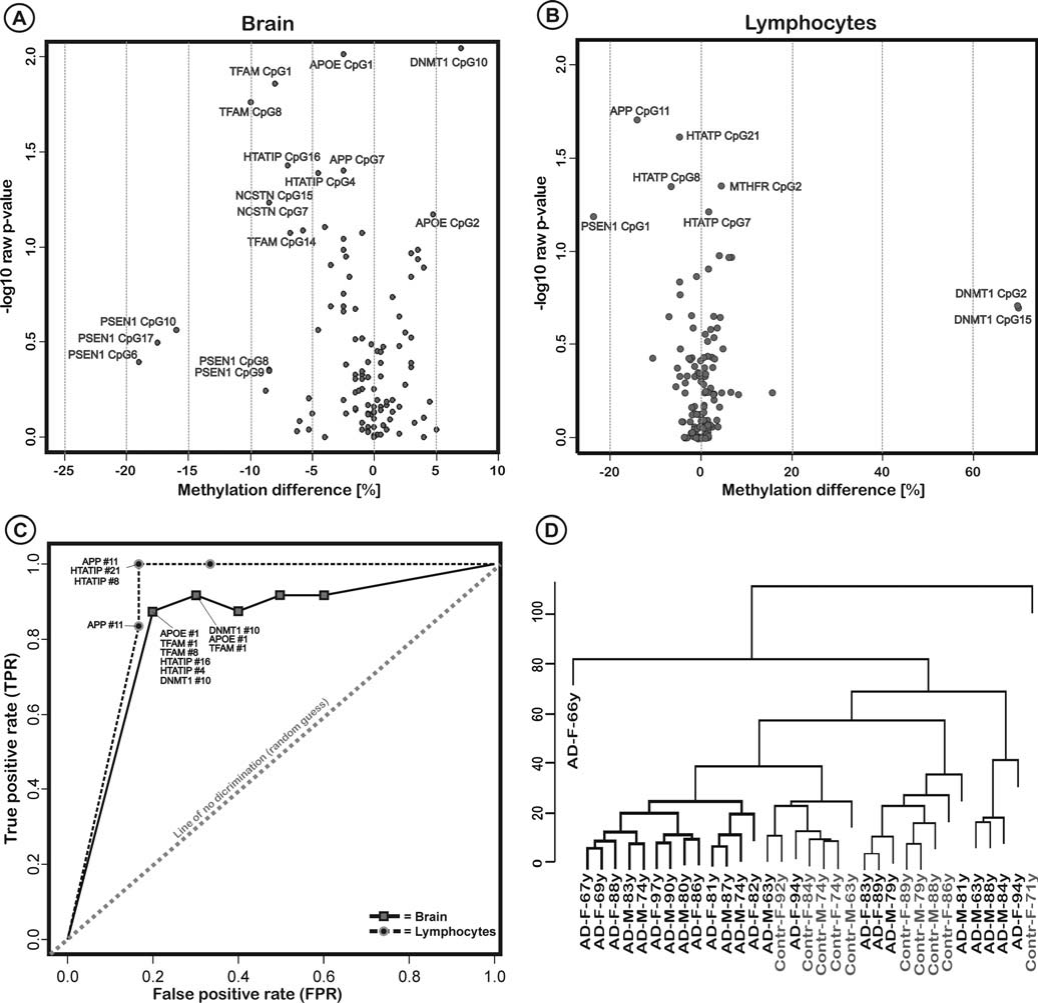
DNA methylation difference between AD cases and controls. A, B: Volcano plots of methylation differences by single CpG sites in brain and lymphocytes. Methylation differences are calculated by the median of LOAD methylation minus the median of the controls. P-values were calculated by two-sample Wilcoxon tests. The strongest disease-specific epigenetic effect in the LOAD brains could be observed for PSEN1; however, low p-values for that gene indicate high interindividual variance. C: Receiver operating characteristic (ROC) curve of several CpG-site combinations, which may be used for disease classification. The ROC curve represents the fraction of true positives (TPR = true positive rate) vs. the fraction of false positives (FPR = false positive rate). TPR claims on the 24 AD brains increase at the cost of FPR claims on the control brains. The ideal scenario is to have a high TPR combined with a low FPR. The diagonal line divides the ROC space in areas of good or bad classification/diagnostic. Points above the diagonal line indicate good classifiers, while points below the line indicate poor ones. Best accuracy brain = 0.85; lymphocytes = 0.92. D: Hierarchical clustering of LOAD patients and controls using the five top brain methylation biomarkers reveals a clustering of a large group of the LOAD samples (see also Supporting Fig. S3).

To identify disease specific epigenetic signatures or classifiers that can be used as markers to characterize or prognosticate LOAD we performed a detailed statistical investigation including Wilcox rank sum test, hierarchical clustering and discriminant analysis of all the analysed CpG sites (see Material and Methods). We found that only a small subset of the CpG sites included in the study were significantly different in the LOAD patients brains compared to control brains (Fig. 5a). The most significant differences were found in the APOE and TFAM promoters (TFAM CpG #1, #6 and #14; APOE CpG #1 and #2), however, these signatures were associated with only small changes in methylation levels (<10%). Other loci, especially a group of several CpG sites within the PSEN1 promoter were associated with stronger methylation differences, however, only in a subset of individuals, which is reflected in the fact that PSEN1 is the most variable of the analysed genes (Figs. 3e and 5a).

It is interesting to note that the changes observed in LOAD patients were usually correlated with a further demethylation of the PSEN1 and TFAM promoters. The analysis of differentially methylated CpG sites in lymphocytes could not reproduce the patterns that we found in the brain samples (Fig. 5b), and also did not reveal clusters of affected CpG sites, but merely single disease specific markers; which may indicate that no specific gene promoters are altered in the blood of LOAD patients. Hence, for the analysed genes, the use of specific blood biomarkers for the diagnostics of LOAD seem unlikely, although these sites can be used to classify the majority of our LOAD cases and controls with high accuracy (Fig. 5c).

Sites for optimal performance of classifier-sets were determined by the technique of receiver-operating-characteristic (ROC) curve. Points in an ROC plot indicate the sensitivities and specificities of a given group of methylation classifiers. In general, true positive claims on the LOAD brains increase at the cost of increasing false positive claims on the control brains. For the brain samples, a combination of several markers was able to classify over 80% of the brain samples correctly as LOAD. Using blood markers, the classification could reach over 90%. However, it is not known if the same classifiers can be successfully applied to other LOAD cases.

Disease classification was complemented with clustering algorithms that may reveal systemic changes in the brains of LOAD patients and that serve as training sets for the potential biomarkers. Clustering of the 34 brains was done by applying a divisive hierarchical clustering algorithm using the top five methylation sites (see Material and Methods). The resulting tree shows that the 5 most significant CpG sites serve well as biomarkers for discrimination, with a clustering of the majority of LOAD samples (Fig. 5d). These data show that epigenetic markers may be suitable to further characterize LOAD and other complex diseases; however more detailed analyses, including a whole genome approach with many samples, are needed.

## Discussion

It is increasingly acknowledged that epigenetic phenomena may be a crucial component in the development of complex brain disorders. Indeed, we and colleagues recently demonstrated that epigenetic mechanisms play an important role in neurobehavioral diseases such as schizophrenia and bipolar disorder [21]. Surprisingly, although many non-Mendelian characteristics point also to an involvement of epigenetic factors in age-related diseases, very little is known about epigenetic patterns in AD and other neurodegenerative disorders. From twin studies we know that the onset of AD in identical twins not only can differ by more than 20 years [22], [23], we also know that young identical twin pairs are essentially indistinguishable in their epigenetic profiles, whereas older twin pairs show substantial differences in their epigenetic marks [24], [25]. These variations can be explained by an epigenetic drift caused by one's environmental exposure, lifestyle, diet, drug abuse, or merely stochastic fluctuations. Conventional wisdom is that the majority of environmental effects are likely to have only a small impact on the aging process and hence, it would be expected that age-specific effects would cause only minor epigenetic changes in a subset of genes. Indeed, we did not find major ‘all or nothing’ DNA methylation changes in the brains of LOAD patients. Nevertheless, the observed methylation drift and the fact that the most unusual methylation patterns were almost always observed in the LOAD brains support the role of epigenetic deregulation in age-dependent AD. Most of the analyzed CGI regions were predominantly unmethylated and it seems plausible that even small changes in the methylation levels of the CGI's interfere with critical gene regulatory functions, potentially accumulating to finally cause the disease. A subset of the analyzed gene promoters (i.e. NCSTN, TFAM, SIN3A or HTATIP) displayed only one or two ‘abnormal’ methylation patterns observed only in the LOAD brains. Yet, since each cell type in the brain possesses a characteristic profile of DNA methylation specific to its function, we cannot rule out that the observed methylation patterns could also be the consequence of a shift in cellular population heterogeneity, associated with loss of specific cell types in the diseased individuals. Shifts in cell heterogeneity were described as ‘one of the major stumbling blocks of biological investigations of aging’ [25]. To address this limitation, future studies on epigenetic drift in the aging brain will require special methodologies such as laser capture microdissection (LCM) and flow cytometry combined with whole-genome single-cell methylome profiling. Nevertheless, the occurrence of abnormal methylation pathways in the brain that precede pathological findings and dementia supports our model that epigenetic drift may be one contributor to LOAD predisposition [26]–[29].

### Interindividual variance

In our study, the largest interindividual variance in DNA methylation was observed in the PSEN1 and APOE promoters, the two genes that are genetically associated with LOAD. In the case of PSEN1, it is known that its promoter is regulated by DNA methylation [16] and may be up- or down-regulated in AD, depending on the cell-type analyzed [30]. In the analyzed LOAD brain samples, abnormal PSEN1 methylation patterns were usually associated with hypomethylation of the promoter. This finding may be important, because hypomethylation could induce an over-expression of PSEN1, which could result in an imbalance in β-amyloid production [31]. Previously, we and colleagues showed that PSEN1 as well as PSEN2 contain epigenetic variability already in male germ cells [32]. Such patterns may be transmitted directly through the germline or may be re-established postzygotically, which may contribute to different susceptibility to disease later in life.

### Bimodal methylation patterns in APOE

We found the APOE promoter region to be one of the most variably methylated sequences. APOE is the major susceptibility gene for late-onset AD in the human genome [33]. Nevertheless, although there are links between APOE genotype (i.e. the APOE ε4 allele) and SNP rs4420638, located 14 kbp distal to APOE, it seems that APOE genotyping has no major role in predicting the risk of developing AD. Even individuals with the rare ε4/ε4 genotype have, on average, a greater than 50% chance of escaping the disease [2], indicating that genetic variants of this allele are not a sufficient cause of AD. Interestingly, as shown in Fig. 2c, although the APOE allele's ε2 and ε4 also change the amino acid sequence of APOE, they also alter the epigenetic information of the methylated downstream CGI, by removal of a CpG dinucleotide. Elimination of one of these methylated CpG units thus may impair the regulatory function of the 3′-CGI. Indeed, the presence of the ε2 or ε4 alleles alone is sufficient to affect the expression patterns of APOE [18]. An even stronger effect on the transcription level of the gene is exerted by the main promoter, which contains numerous *cis*-acting positive and negative regulatory elements [34], although the CpG density of the APOE promoter is comparably low.

### Epigenetic drift and age-effects

For all of the analyzed CGI's, the absolute epigenetic difference from the healthy norm was higher in LOAD brains compared to the controls. Although these changes were only small, they may indicate a genome wide epigenetic drift in LOAD brains that is accompanied by a slighter increased variance with age in the LOAD brains but not in the controls. It is important to note that it is unlikely that age-dependent epigenetic drift will manifest itself by switching AD susceptibility genes completely on or off, especially if the majority of changes are due to stochastic fluctuations, which could be more common than is generally assumed [35]. In contrast to single nucleotide polymorphisms (SNPs), epigenetic modifications may exert only subtle effects on the regulation of specific genes. Thus, abnormal DNA methylation may only cause a disease phenotype when several loci are affected at the same time, reflected as epigenetic distance to the norm, or by increasing the predisposition to AD of affected individuals, which are already prone to develop AD by other predisposing factors such as APOE genotype or SNPs (2-hit hypothesis). We observed the strongest epigenetic distance in patient brains for the TFAM promoter. Mutations in TFAM, which is required for mitochondrial DNA copy number regulation and maintenance, may be a moderate risk factor in sporadic AD. Although the disease specific pattern methylation markers within TFAM may help characterizing the disease (Fig. 3c), the measured profiles within TFAM alone are not powerful enough to diagnose LOAD.

Since the main characteristic of LOAD is the late age of onset, we looked for age-effects in the DNA methylation profiles. The risk of developing the disease doubles every 5 years over age 65 and may affect up to half the people older than 85. If cumulative stochastic or environmental epigenetic insults do affect disease development it is to be expected that the epigenetic distance from the norm increases progressively with age. This is indeed what we observed for the 24 LOAD brains. An age-dependent epigenetic drift was also observable in lymphocytes from patients and controls (Supporting Fig. S1), however the effect was significantly smaller compared to the brain tissues. The strongest age-effects were detected in the NCSTN gene that codes for nicastrin, which participates in the regulation of γ-secretase cleavage of the amyloid precursor protein. The affected neuronal tissues in the AD brains may be prone to collect epimutations with time due to their post-mitotic state. In contrast, it can be hypothesized that cells which are constantly renewed can repair epimutations much more efficiently. Indeed, only a minor age-specific epigenetic drift could be identified for the lymphocytes in our samples, although we analyzed just a limited number of samples.

Age-dependent methylation changes associated with AD have been previously reported for the APP gene. Toghi *et al*., reported that some of the CpG sites within the APP promoter can be partially methylated in brains of healthy individuals (∼26% methylation), accompanied by a reduction with age (<8%) in methylcytosine content in these CpG sites [36]. However, in contrast to the previous study, using a more sensitive technique we could not replicate any age-specific effects in our brain samples, where APP seemed to be stably hypomethylated (∼20%) throughout old age in healthy subjects and LOAD cases. The discrepancy may be largely due to the limited sample size and sensitivity of the older study. Interestingly, it could be shown that the APP gene can be monoallelically expressed at certain stages in a subset of individuals [6]. A person with such “wrongly” expressed gene might be increasingly affected by epigenetic deregulation, resulting in a higher susceptibility to an earlier onset of AD [37].

It is known that some DNA methylation patterns can change with aging progressively in a complex, cell-type specific fashion [24], [38]. Although several reports showed that an overall decrease in methylcytosine content with aging in many vertebrate tissues is a common phenomenon (reviewed in [39]), in our brain and lymphocyte samples we did not observe any significant progressive change in methylation levels.

### Epigenetic theory of LOAD

Many studies demonstrated that epigenetic alterations occur in higher frequency than genetic mutations and could, thus, be particularly important in age-related phenotypes [40], [41]. The high frequency of de novo epimutations suggests that epigenetic alterations accumulate during ageing. Small epimutations in the critical genes may be tolerated to a certain degree and merely reflect the range of interindividual variance. However, once a critical threshold of epigenetic deregulation is reached, the brain starts to malfunction. In this regard, LOAD may represent merely an extreme form of normal aging, which would imply that every human being has a certain predisposition to develop Alzheimer's. In our model, the epigenetic effects can accumulate throughout life, especially from the time-point when the epigenetic machinery suffers from old age, but also from early embryonal stages or even trans-generational, influenced by epigenetic events in the parents. A particularly illustrative example of environmental effects was reported by Basha et al., which demonstrated that exposure of rats to lead (Pb) from birth to postnatal day 20 showed a delayed overexpression of APP and elevation of its amyloidogenic Aβ production in old age [42]. Similarly, it was reported that the expression of APP and BACE were elevated in aged cynomolgus monkeys that were exposed to Pb as infants, implicating that environmental agents may play a role in the pathogenesis of AD [43]. Support for the idea that LOAD may merely represent an extreme form of aging or age-dependant epigenetic drift comes from a system level analysis of transcriptional changes in AD and normal aging [44]. Strikingly, it was found that specific genes not only show remarkable parallel expression changes in AD and aging, but many also cluster into modules within a transcriptional coexpression network related to synaptic and mitochondrial function, supporting the notion that LOAD and normal aging share common pathophysiological processes.

Several additional phenomena exist that support an epigenetic model of LOAD development (see also Supporting Text S1). For example, concordance rates of monozygotic (MZ) twins are far from 100% and were estimated from 21% to 83%. If the major disease causing factors were genetic, higher concordance rates would be expected. Furthermore, the onset of AD between MZ twins who eventually even became concordant often differs more than 2 decades [23], further supporting the effect of either harmful or protective environmental or epigenetic factors playing a significant role in the occurrence of AD.

Another typical sign of epigenetic deregulation is the occurrence of gender effects; however we observed only minor abnormalities within the DNMT1 promoter in male LOAD brains. These results are in agreement with previous data from the human epigenome project [12], which did not detect any significant methylation differences between sexes in 873 analyzed genes.

Another typical epigenetic abnormality observed in LOAD patients is an abnormal one-carbon methylation metabolism, indicative by elevated plasma homocysteine (Hcy) and low serum folate concentrations. Both Hcy and folate are critical components of a series of biosynthetic pathways essential for DNA and histone methylation reaction. Epidemiological studies have shown that elevations in plasma Hcy temporally precede the development of dementia and that there is an inverse linear relation between plasma Hcy concentrations and cognitive performance in older persons [26]–[29]. These observations could indicate that some epigenomic pathways are disrupted before the main LOAD phenotypes, such as the forming of amyloid plaques, occur. It was also reported that S-Adenosymethionine (SAM), which is required for the methylation of DNA as well as methylation of histones, is severely decreased in the spinal fluid and brains of AD patients [45], [46]. It is interesting to note that total Hcy levels are not only increased in AD patients compared to controls, but also significantly increased in LOAD compared to early-onset AD (EOAD) [47], indicating that in both AD subtypes two very different events are taking place, which eventually lead to the same AD phenotypes.

Another component of the methylation pathways is the methylenetetrahydrofolate reductase (MTHFR) that catalyzes the conversion of 5,10-methylenetetrahydrofolate to 5-methyltetrahydrofolate, a co-substrate for homocysteine remethylation to methionine. An extreme deficiency of MTHFR results in severe hyperhomocysteinemia and brain abnormalities and may be associated with a decrease of global DNA methylation level. In our brain samples the MTHFR promoter displayed a high interindividual variance in DNA methylation. Some extreme methylation levels may, in addition to known polymorphisms that are known to influence Hcy levels, be involved in the observed elevated Hcy levels in AD patients.

Another, not very well understood phenomenon in AD is the occurrence of chromatin abnormalities and aberrant histone regulation. Using the cytosine nucleoside analogue and antileukemic drug 5-azacytidine (5-AzaC) on cultured lymphocytes, it was shown that AD patients have a significantly increased frequency of undercondensation of constitutive heterochromatin of chromosomes 1, 9 and 16, when compared with control groups [48]. This study identified an age-dependent 5-AzaC sensitive region on chromosome 19q13 encompassing the APOE gene, which may indicate that with increased age, the chance of chromosomal abnormalities increases. It is likely that the epigenetic patterns in the cells serve as long-term memory storage [49]. Epigenetic drift would interfere with this information storage and it could be expected that some neurons would lose their epigenetic memory, resulting in mitotic competence and cell cycle reactivation, a phenomenon that was observed in vulnerable regions of AD brains, but not control brains [50].

In summary, the data from our study suggest that in addition to genetic determinants and environmental effects, an important factor in occurrence of LOAD is epigenetic variability. Epigenetic drift and interindividual DNA methylation profiles may affect LOAD predisposition and course of disease. Instead of single biomarkers, epigenetic drift may be a suitable marker for disease predisposition. More studies are needed to address this possibility.

## Materials and Methods

### DNA-Samples

Post-mortem brain tissues derived from the prefrontal cortex of individuals with diagnosed AD (n = 24) and matched controls (n = 10) were provided by the Department of Neuropathology at the Ludwig-Maximilian University in Munich. The samples consisted of frozen tissue sections, which were stored at −80°C prior to nucleic acid extraction. Lymphocyte samples with diagnosed AD (n = 6) and matched controls (n = 6) were collected at the Klinikum rechts der Isar, where the control group was matched for geographical location, ethnicity, sex and age and consisted of cognitively healthy individuals (for details see Supporting Material & Methods S1).

### Bisulfite treatment of genomic DNA

Bisulfite treatment was performed using a standard protocol as described by Clark and colleagues [51]. Briefly, ∼500 ng genomic DNA was denatured in 0.3 M NaOH for 15 min at 37°C. After adding freshly prepared 3.5 M sodium metabisulfite (Sigma) and 1 mM Hydroquinone (Sigma) solution, samples were subjected to 4-hour incubation at 55°C under exclusion of light. The samples were then purified using Qiagen DNA purification columns (Qiagen). Recovered samples were desulfonated in 0.3 M NaOH for 15 min at 37°C and neutralized. DNA was precipitated overnight in ethanol at −20°C and resuspended in 50 µl buffer EB (Qiagen). Bisulfite treated DNA was stored at −80°C until needed.

### Primer design and MS measurements

A full list of primer sequences and annealing temperatures for each PCR reaction can be found in Supporting Table 1. PCR amplifications were performed using a standard hot-start PCR protocol in 20 µl volume reactions containing 2 µl of sodium-bisulfite treated DNA. All PCR reactions were checked on a 1.0% agarose gel to ensure successful amplification and specificity before proceeding with the MALDI. For the MALDI analysis, bisulfite-PCR products were processed according to the manufacturer's standard protocol (Sequenom, USA). Detailed PCR and in vitro transcription conditions are described in the Supporting Material & Methods S1. The cleavage reactions (20–25 nl) were robotically dispensed onto SpectroCHIP silicon chips preloaded with matrix (Sequenom). Mass spectra were collected using a MassARRAY® Compact Analyzer and a Bruker Autoflex mass spectrometer. Spectra were analyzed using the EpiTYPER (Sequenom) software tool.

### Data analysis

#### Comparison of DNA methylation distances across individuals

The distribution of the methylation intensities at a specific site in a group of individuals does not follow a Gaussian distribution; hence to identify differentially methylated sites between cases and controls, we used the Wilcox rank sum test for the null hypothesis of equal distributional locations (medians). The p-value returned from the test is −log_10_ transformed and serves as the y-coordinate; the difference in the medians input to the test serves as the x-coordinate. The test was run site by site, resulting in the ‘volcano’ plot. Top corners of the plot encompass sites that are significantly different in methylation between the two groups (either AD vs. control or male vs. female). Adjustment of p-values for multiple testing was performed by the Bonferroni method since the number of sites is not large.

#### Epigenetic distance

The deviation *d* of the methylation profile of an affected individual to the group of unaffected controls is measured by the Euclidean distance,
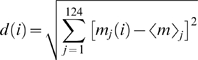
where *m_j_*(*i*) is the methylation intensity at site *j* of affected individual *i* and <*m*>*_j_* is the median methylation of the controls at site *j*. The larger the *d*(*i*), the more different individual *i* is from the group of controls. We then calculated the Pearson product-moment correlation coefficient between the AD patients' methylation deviates *d*'s and their ages. The p-value associated with the correlation coefficient indicates the probability of the correlation being due to chances alone. For a detailed description of hierarchical clustering and principal component analysis see Supporting Material & Methods S1.

### Web resources

The DNA methylation data presented herein can be accessed at our Epigenetics database under this URL: http://www.methylogix.com/genetics/database.shtml.htm

## Supporting Information

Materials and Methods S1 (0.05 MB DOC)

Figure S1 Age-specific epigenetic drift in human lymphocytes. The epigenetic Euclidean distance of 12 lymphocyte samples compared to the ‘epigenetic norm’ increases with age (r = 0.24; p-value = 0.46), however less increase compared to the LOAD brain samples.
(0.30 MB TIF)

Figure S2 Age-specific DNA methylation in brains. Despite a significant epigenetic drift, represented by demethylation and de novo methylation of gene promoters with age in individuals with late-onset AD patients, most genes retained an average methylation with ongoing age.
(1.23 MB TIF)

Figure S3 Principal component analysis (PCA) of 34 brain samples using the five most significant CpG sites. Black circles = LOAD brains; orange circles = control brains. A further visualization of the epigenetic relationship of the brain samples in relation to the identified top LOAD markers using principle component analysis (PCA) also demonstrated a clustering and hence similarity of a substantial part of the LOAD samples. A: Three dimensional PCA. B: Biplot PCA. Two neighboring points indicate two similar brains in terms of the five sites. Two neighboring lines indicate high correlation between the two sites among the brains. Points along a line indicate brains that vary in methylation at the site. The roughly even spacing of the five lines in the resulting biplot indicates that the five selected sites work synergistically in distinguishing the brains.
(0.60 MB TIF)

Text S1 Supporting text file
(0.09 MB DOC)

Table S1 (0.08 MB DOC)

Table S2 (0.04 MB DOC)
