# Supplementary material for: Age-Specific Epigenetic Drift in Late-Onset Alzheimer's Disease
Source: PLoS One. 2008 Jul 16;3(7):e2698. doi: 10.1371/journal.pone.0002698 (PMC2444024; doi:10.1371/journal.pone.0002698)
Supplement: Materials and Methods S1 — (0.05 MB DOC) [file pone.0002698.s001.doc]

**Supporting Materials & Methods**

**DNA/Patients**

Frozen brain tissues (prefrontal gyrus frontalis superior) from randomly selected control individuals (n=10, mean age at death 80.0±9.8 years) and AD patients (n=24, mean age at death 80.9±9.3 years) with clinically and neuropathologically confirmed late-onset AD was obtained from the Departments of Neuropathology at the Ludwig-Maximilians University in Munich and the Technical University Munich. Blood samples were obtained from patients with LOAD (n=6, mean age 81±4.5 years) and age-matched controls (n=6, mean age 80.0±5.2 years), who were recruited at the university dementia outpatient unit of the Dept. of Psychiatry, TU-Munich. The patient samples had a female to male ratio of 0.5. The control group was matched for gender, age, geographical location and ethnicity and consisted of cognitively healthy subjects. The study protocol was approved by the institutional review board of the universities. The clinical diagnosis of probable AD was established according to National Institute of Neurological and Communicative disorders and Stroke-Alzheimer's Disease and Related Disorder Association (NINCDS-ADRDA) criteria [1]. After informed consent had been obtained, blood samples of each individual were taken by venous puncture. Cognitive performance was assessed using standard neuropsychological tests, such as the Consortium to Establish a Registry for Alzheimer's disease, which includes the Mini Mental State Examination (MMSE) [2], which was used as a measure of global cognitive performance in the control groups. Control subjects with an MMSE score below 28 were excluded from further analyses. All patients and controls underwent a thorough psychiatric, neurological and neuropsychological evaluation. Extraction of DNA was performed using a standard salt and phenol/chloroform extraction method, following overnight tissue digestion with proteinase K. The quality and quantity of DNA was assessed by spectrophotometry and agarose gel analysis, and subsequently stored at -20°C until further use.

**MALDI-TOF mass spectrometry analysis**

In this method, an initial PCR amplification of the target region, usually a CpG island (CGI) from bisulfite-treated DNA is followed by an *in vitro* transcription to generate single-stranded RNA, which is then cleaved base-specifically. Cleavage occurs either after C or U, determined by the usage of non-cleavable nucleotides [3]. Since the bisulfite treatment introduces C 🡪 T conversions in the DNA (reflected as G/A changes on the reverse strand), the cleavage reactions result in a mass difference of 16 kDa for each CpG site enclosed in the cleavage products generated from the RNA transcripts that can be analyzed using MS. The intensities of the signals for methylated and unmethylated fragments are compared, and the relative amount of methylated DNA is calculated from the ratio of the signal pairs of cleaved and non-cleaved fragments. The method yields quantitative methylation levels for each of these fragments, which contain either one individual CpG dinucleotide or an aggregate of several adjacent CpG sites, which are defined as ‘CpG units’. To avoid technical errors and to increase accuracy, measurements were done for the T- and C-cleavage reactions, both on two different mass spectrophotometers, so the resulting values are the average of 4 measurements.

**PCR and in vitro transcription**

The target regions were amplified using the primer pairs described in Supplementary table 1. A typical PCR was carried out in a total volume of 20 µl using approx. 1.4 µM of each primer, 0.5 µl of 25mM deoxynucleotide triphosphate (dNTP), 2 units HotStar Taq DNA polymerase (Qiagen, Hilden, Germany), 1.5 µl of 25 mM MgCl_2_, and buffer supplied with the enzyme (final concentration, 1x). Usually, primer annealing temperature, number of cycles and Mg concentrations were optimized for individual primer pairs. For detailed PCR conditions for a specific primer pair, contact the authors. After PCR (and nested PCR), 5 µl of the product were used for further reactions. Unincorporated dNTPs were dephosphorylated by adding 1.7 µl H_2_O and 0.3 units shrimp alkaline phosphatase (SAP; SEQUENOM, Inc., San Diego, CA). The reaction was incubated at 37°C for 20 minutes and SAP was then heat inactivated for 5 minutes at 85°C. Typically, 2 µl of the reaction mixture were directly used as template in a 7 µl transcription reaction. Twenty units T7 R&DNA polymerase (Epicentre, Madison, WI) were used to incorporate either dCTP or dTTP in the transcripts. Ribonucleotides were used at 1 mmol/l and the dNTP substrate at 2.5 mmol/l. Other components in the reaction were as recommended by the supplier. In the same step, the in vitro transcription RNase A (Sequenom) was added to cleave the in vitro transcript. The mixture was then further diluted with H_2_O to a final volume of 27 µl. Conditioning of the phosphate backbone before MALDI-TOF MS was achieved by the addition of 6 mg Clean Resin (Sequenom). Further experimental details have been described elsewhere [4,5].

**Hierarchial clustering and Principle component analysis**

Due to the number of brains it would be inefficient to allocate a subset of the brains for training the classifier and the rest for validation of the prediction. Instead, we employed the leave-one-out-cross-validation scheme for training and validating the k-nearest-neighbor (kNN) classifier, which can be easily used for smaller sample-sizes. The kNN in our setting works in the following fashion:

The training set consists of 34 brains whose classes (LOAD or controls) are known

- For brain i, its Euclidean distances to the other 33 brains are calculated
- The k brains which are closest to brain i are identified
- The class of brain i is then determined by the majority of the k brains’ classes

Not all methylation sites are informative in discriminating LOAD’s from controls; however the volcano plots provided clues. Furthermore, in general, true positive claims on the LOAD brains increase at the cost of increasing false positive claims on the control brains. The value of k and informative sites for optimal performance of the classifier was determined by the technique of receiver-operating-characteristic (ROC) curve. Points in an ROC plot indicate the sensitivities and specificities of the classifier at various conditions. The clustering of the 34 brains was done by the divisive hierarchical clustering algorithm using the top five methylation sites from the volcano plot of Fig. 5a. Divisive hierarchical clustering is good at coarse partitioning and is suitable for the present application as we know there are only two groups (LOAD and controls) in the brains. The resulting tree shows that the five chosen cytosine sites serve well as biomarkers for discrimination.

Visualization of the brain data was done by principle component analysis (PCA), where the five selected sites represent five variables (or factors) and the 34 brains as 34 independent observations (or realizations) of the five variables. PCA is a transformation of the five dimensional dataset into a two or three dimensional space while preserving most of the correlation in the dataset. A biplot of the PCA result is a presentation which enables visualization of the brain-brain, site-site, and brain-site relations in a single plot (see supplementary figure 3).

**References**

1. McKhann G, Folstein M, Katzman R, Price D, Stadlan EM (1984) Clinical diagnosis of Alzheimer's disease: Report of the NINCDS-ADRDA work group under the auspices of Department of Health and Human Services Task Force on Alzheimer´s Disease. Neurology 34: 939-944.

2. Folstein MF, Folstein SE, McHugh PR (1975) 'Mini Mental State'. A practical method for grading the cognitive state of patients for the clinician. J Psychiat Res 12: 189-198.

3. Hartmer R, Storm N, Boecker S, Rodi CP, Hillenkamp F, et al. (2003) RNase T1 mediated base-specific cleavage and MALDI-TOF MS for high-throughput comparative sequence analysis. Nucleic Acids Res 31: e47.

4. Ehrich M, Field JK, Liloglou T, Xinarianos G, Oeth P, et al. (2006) Cytosine methylation profiles as a molecular marker in non-small cell lung cancer. Cancer Res 66: 10911-10918.

5. Ehrich M, Nelson MR, Stanssens P, Zabeau M, Liloglou T, et al. (2005) Quantitative high-throughput analysis of DNA methylation patterns by base-specific cleavage and mass spectrometry. Proc Natl Acad Sci U S A 102: 15785-15790.
