# Supplementary figures and images for: Age-Specific Epigenetic Drift in Late-Onset Alzheimer's Disease
Source: PLoS One. 2008 Jul 16;3(7):e2698. doi: 10.1371/journal.pone.0002698 (PMC2444024; doi:10.1371/journal.pone.0002698)

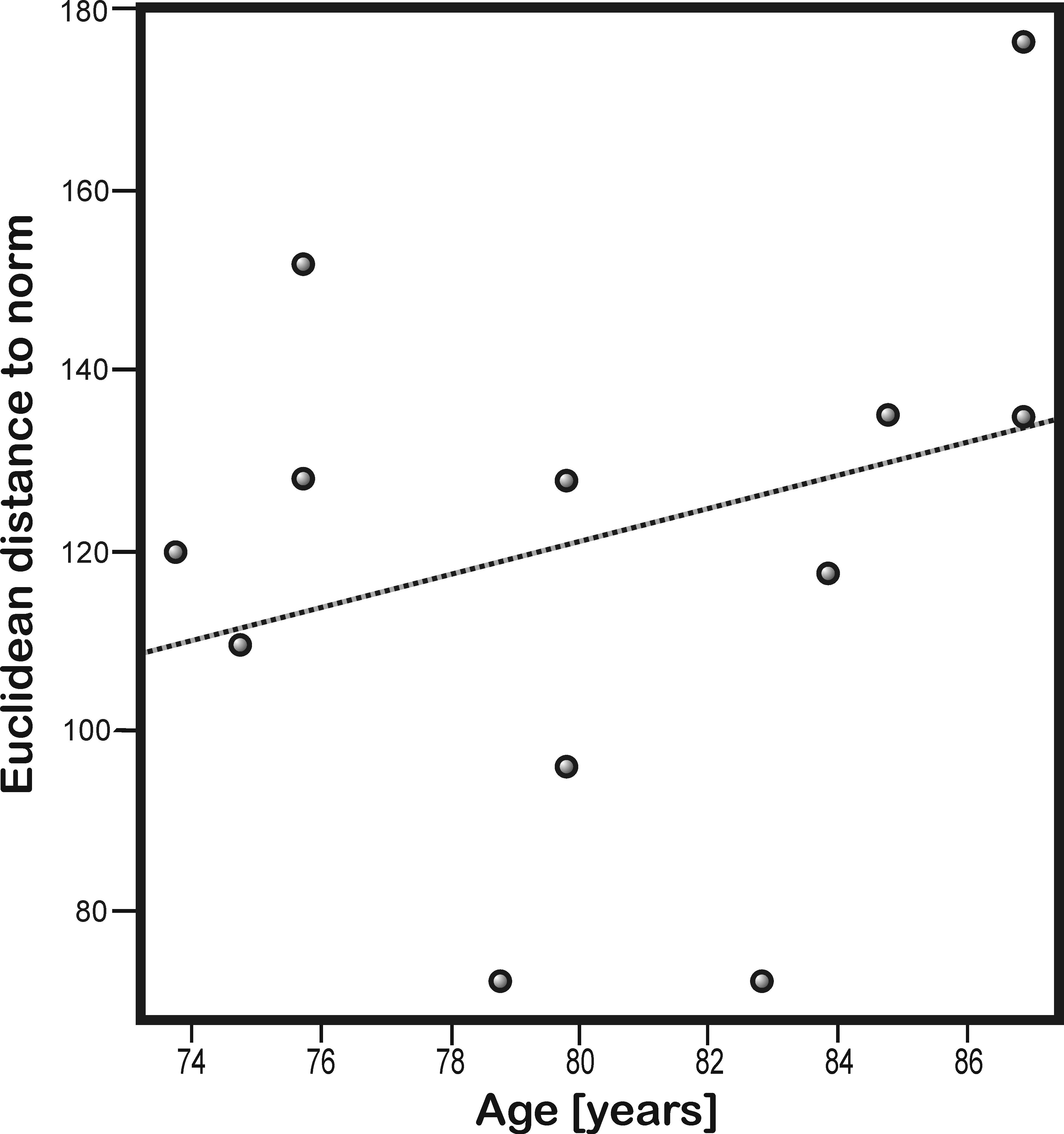

Supplement: Figure S1 — Age-specific epigenetic drift in human lymphocytes. The epigenetic Euclidean distance of 12 lymphocyte samples compared to the ‘epigenetic norm’ increases with age (r = 0.24; p-value = 0.46), however less increase compared to the LOAD brain samples. (0.30 MB TIF) [file pone.0002698.s002.tif]

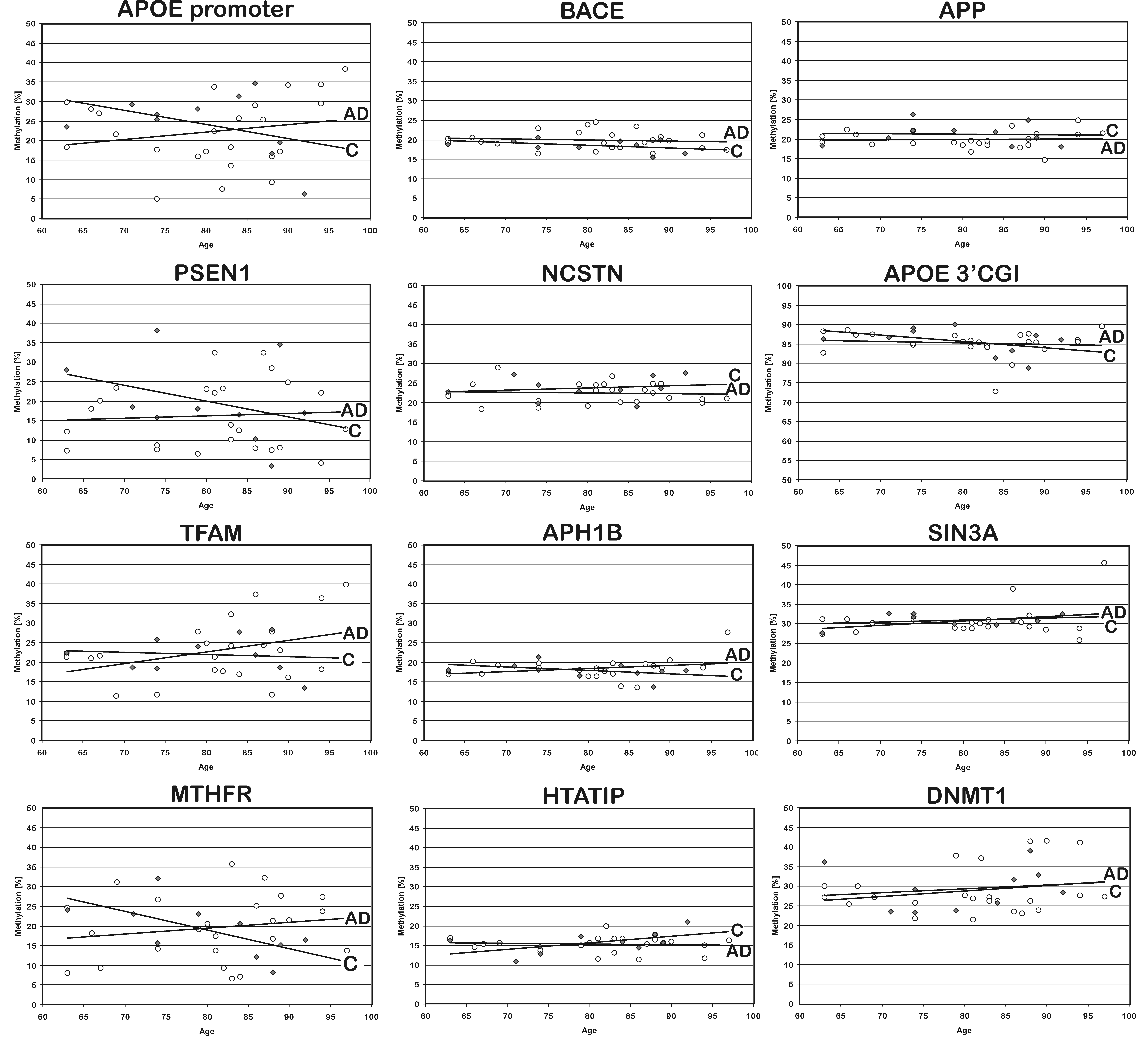

Supplement: Figure S2 — Age-specific DNA methylation in brains. Despite a significant epigenetic drift, represented by demethylation and de novo methylation of gene promoters with age in individuals with late-onset AD patients, most genes retained an average methylation with ongoing age. (1.23 MB TIF) [file pone.0002698.s003.tif]

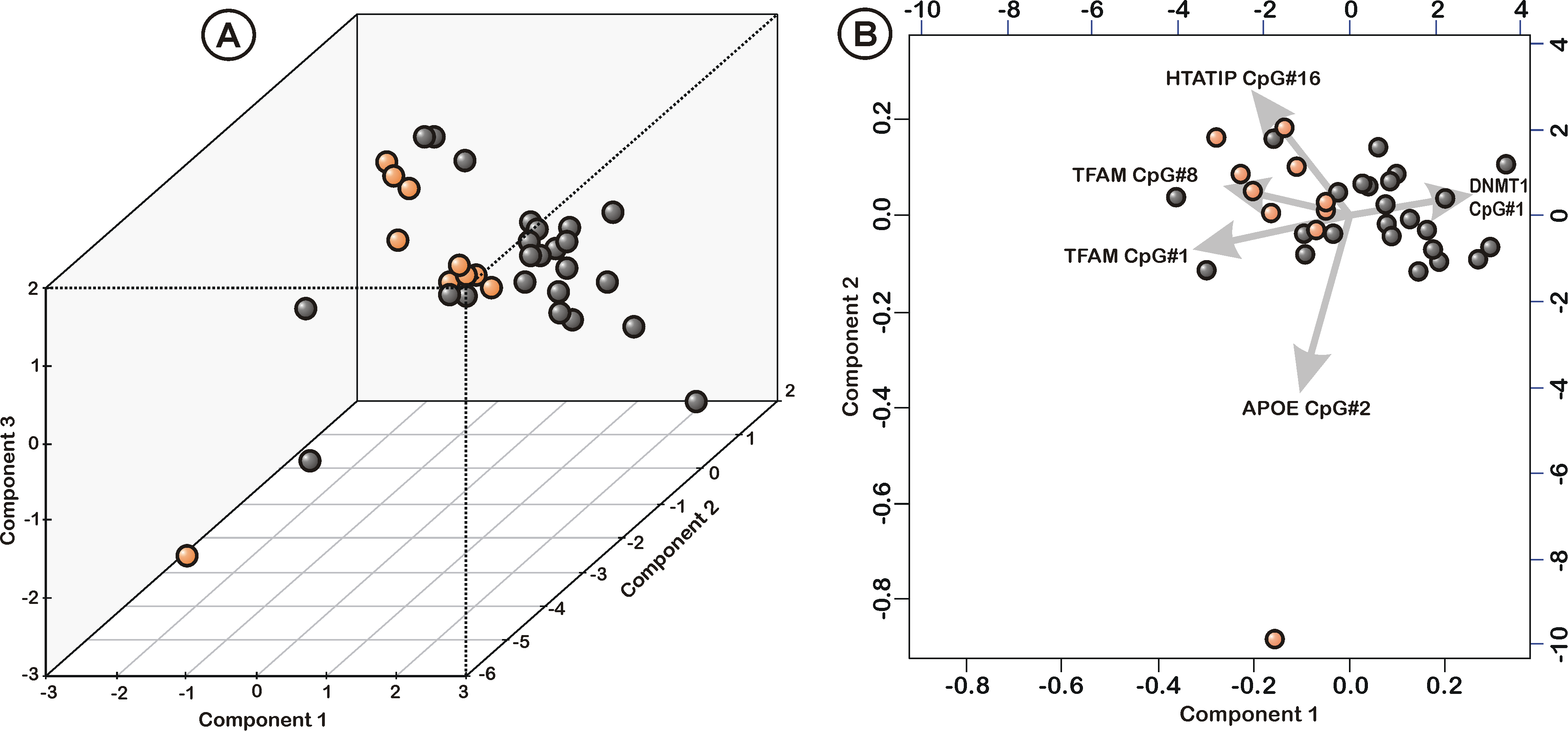

Supplement: Figure S3 — Principal component analysis (PCA) of 34 brain samples using the five most significant CpG sites. Black circles = LOAD brains; orange circles = control brains. A further visualization of the epigenetic relationship of the brain samples in relation to the identified top LOAD markers using principle component analysis (PCA) also demonstrated a clustering and hence similarity of a substantial part of the LOAD samples. A: Three dimensional PCA. B: Biplot PCA. Two neighboring points indicate two similar brains in terms of the five sites. Two neighboring lines indicate high correlation between the two sites among the brains. Points along a line indicate brains that vary in methylation at the site. The roughly even spacing of the five lines in the resulting biplot indicates that the five selected sites work synergistically in distinguishing the brains. (0.60 MB TIF) [file pone.0002698.s004.tif]
