# Supplementary material for: Age-Specific Epigenetic Drift in Late-Onset Alzheimer's Disease
Source: PLoS One. 2008 Jul 16;3(7):e2698. doi: 10.1371/journal.pone.0002698 (PMC2444024; doi:10.1371/journal.pone.0002698)
Supplement: Text S1 — Supporting text file (0.09 MB DOC) [file pone.0002698.s005.doc]

**Supplementary information**

Presented is a list of additional epigenetic phenomena that may be important in LOAD predisposition.

1. SIN3A and HTATP are not only interesting for their association with AD, furthermore both participate themselves in chromatin remodeling. For example, cleavage of APP also results in production of an intracellular fragment, the APP intracellular domain, which serves as transcription factor that recruits HTATP to chromatin, which in turn affects histone acetylation. Similarly, the transcriptional regulator SIN3A as a component of a histone deacetylase complex and by interaction with histone deacetylase HDAC1 directly affects histone acetylation.
2. PSEN1 also regulates the transcription of the cAMP response element-binding protein (CREB)-CREB binding protein (CBP) pathway, a signaling mechanism that is important for neuronal survival. CBP is itself part of the epigenetic machinery of the cell since it possesses a histone acetyltransferase function that is important for long-term memory formation and that influences higher order chromatin structure [1].
3. Another study using Southern-blot analysis demonstrated the absence of DNA methylation at a HpaII site in the APP gene of an AD subject, which was methylated in a control individual [2], though the analysis of one AD case compared to one control can merely demonstrate that interindividual differences exist. Yet, various cis-acting regulatory elements located in 5' distal regions are known to play a main role DNA methylation levels at the APP promoter and were shown to exhibit gender differences in mice [3]. However, no sex-specific differences could be identified in our human samples.
4. Using a mouse model, it was demonstrated that levels of DNA methylation in brain increased upon ischemic injury, and this increase was partly dependent on Dnmt1 activity [4]. Furthermore, it was shown that environmental enrichment reinstalled learning behaviour in mice and re-established long-term memory after significant brain atrophy, a process that correlated with chromatin modifications (i.e. increased histone acetylation) [5]. In this context, it is highly interesting to note that head injury is one of the most potent environmental risk factors for subsequent development of AD. It is possible that a head injury doesn't induce AD-like pathology so much as accelerates its onset. Moreover, it seems that AD rates are not elevated among subjects with a history of head trauma, but that the injury accelerates the time to AD onset by several years. These phenomena can be explained with the epigenetic theory of LOAD.
5. The occurrence of gender effects is another sign of epigenetic mechanisms. Despite some contradictory studies on the difference prevalence of AD among men and women, the majority of epidemiological studies suggest that women are at increased risk of AD. It can be hypothesized that differential susceptibility to AD in males and females is mediated by sex hormone-induced differences in the epigenetic regulation of certain genes. In accordance with this theory, it was shown that estrogen attenuates AD symptoms in postmenopausal women [6] and that estrogen plays generally a protective role in the time of onset and occurrence of AD [7-9].
6. Other epigenetic abnormalities in LOAD include chromatin abnormalities in the brain. With more severe forms of AD, patients display a malfunctioning of the circadian clock, which resides in the pacemaker neurons of the hypothalamic suprachiasmatic nucleus (SCN) and seems to be regulated by dynamic chromatin remodeling [10]. The promoter regions of these genes display rhythmic, circadian related changes in histone phosphorylation, methylation and acetylation [11,12]. Functional disruption of the master clock is observed from the earliest AD stages onward and may thus provide valuable information about the very first AD stages and could serve as an indicator of early epigenetic deregulation.
7. In AD patients the levels of folate in the spinal fluid are significantly lower compared to healthy individuals [13]. Folate is a critical cofactor in the conversion of methionine to SAM, hence a folate deficiency will promote SAM depletion and widespread hypomethylation. It was speculated that the lower availability of SAM could be related to the altered expression of genes involved in amyloid precursor protein metabolism, ultimately leading to the accumulation of beta-amyloid in the brain [14]. Similar to PSEN1, BACE (β-secretase), which showed moderate age-related effects in our study, is regulated by DNA methylation [15].
8. Parent-of-origin effects may also indicate epigenetic components in complex diseases; however, data on the parent-of-origin transmission of AD are very controversial. Several studies reported an excess of maternal transmission of AD supporting the theory of maternal parent-of-origin effects in LOAD [16], whereas a preferential paternal transmission seems to be associated with early-onset AD [17].
9. A possible mechanism involved in cell cycle activation could be the aberrant modification of histones, which in turn could lead to chromatin rearrangement in the affected cells. Abnormal histone distributions or modifications were repeatedly observed in AD pathogenesis. For example, it was found that phosphorylated histone H3, a key component involved in chromosome compaction during cell division is increased in hippocampal neurons in AD [18]. Similarly to histone H3, a non-nuclear CNS form of histone H1 was shown to be upregulated in neuronal and astrocytic cells in regions of pathology in AD brains [19].
10. DNA methyltransferase 1 (DNMT1) plays an essential role in maintenance of the global methylation status and evidence suggest that DNMT1 contributes to age-dependent changes in methylation levels [20]. In our samples, some CpG sites within the DNMT1 promoter displayed an age-specific epigenetic drift and high interindividual variation. Interestingly, disease specific methylation patterns in the DNMT1 promoter were nearly exclusively observed in male LOAD brains. A progressive loss of DNMT1 efficacy may induce a global loss of DNA methylation.
11. Many environmental factors were associated with AD, such as toxicological exposure, late maternal age at birth, smoking, high calorie intake, hormonal factors and a reduced level of intellectual and physical activities in midlife. It can be hypothesized that environmental insults leave an ‘epigenetic footprint’ in the non-dividing cells of the brain. Much more studies in cross-cultural comparison of cognitive impairment with age are needed to understand how the environment (i.e. diet and behavioural circumstances) affect the development of dementia. Nevertheless, the “western-lifestyle” may be an essential component in the development of late-onset AD. The situation may be similar to other complex diseases such as cancer, where many migration studies have shown that cancer incidence changes on migration, pointing to a predominant environmental contribution to disease causation [21].

References

1. Korzus E, Rosenfeld MG, Mayford M (2004) CBP histone acetyltransferase activity is a critical component of memory consolidation. Neuron 42: 961-972.

2. West RL, Lee JM, Maroun LE (1995) Hypomethylation of the amyloid precursor protein gene in the brain of an Alzheimer's disease patient. J Mol Neurosci 6: 141-146.

3. Mani ST, Thakur MK (2006) In the cerebral cortex of female and male mice, amyloid precursor protein (APP) promoter methylation is higher in females and differentially regulated by sex steroids. Brain Res 1067: 43-47.

4. Endres M, Meisel A, Biniszkiewicz D, Namura S, Prass K, et al. (2000) DNA methyltransferase contributes to delayed ischemic brain injury. J Neurosci 20: 3175-3181.

5. Fischer A, Sananbenesi F, Wang X, Dobbin M, Tsai LH (2007) Recovery of learning and memory is associated with chromatin remodelling. Nature 447: 178-182.

6. Tan ZS, Seshadri S, Beiser A, Zhang Y, Felson D, et al. (2005) Bone mineral density and the risk of Alzheimer disease. Arch Neurol 62: 107-111.

7. Nee LE, Lippa CF (1999) Alzheimer's disease in 22 twin pairs--13-year follow-up: hormonal, infectious and traumatic factors. Dement Geriatr Cogn Disord 10: 148-151.

8. Tang MX, Jacobs D, Stern Y, Marder K, Schofield P, et al. (1996) Effect of oestrogen during menopause on risk and age at onset of Alzheimer's disease. Lancet 348: 429-432.

9. Wickelgren I (1997) Estrogen stakes claim to cognition. Science 276: 675-678.

10. Crosio C, Cermakian N, Allis CD, Sassone-Corsi P (2000) Light induces chromatin modification in cells of the mammalian circadian clock. Nat Neurosci 3: 1241-1247.

11. Nakahata Y, Grimaldi B, Sahar S, Hirayama J, Sassone-Corsi P (2007) Signaling to the circadian clock: plasticity by chromatin remodeling. Curr Opin Cell Biol 19: 230-237.

12. Etchegaray JP, Lee C, Wade PA, Reppert SM (2003) Rhythmic histone acetylation underlies transcription in the mammalian circadian clock. Nature 421: 177-182.

13. Seshadri S, Beiser A, Selhub J, Jacques PF, Rosenberg IH, et al. (2002) Plasma homocysteine as a risk factor for dementia and Alzheimer's disease. N Engl J Med 346: 476-483.

14. Scarpa S, Fuso A, D'Anselmi F, Cavallaro RA (2003) Presenilin 1 gene silencing by S-adenosylmethionine: a treatment for Alzheimer disease? FEBS Lett 541: 145-148.

15. Fuso A, Seminara L, Cavallaro RA, D'Anselmi F, Scarpa S (2005) S-adenosylmethionine/homocysteine cycle alterations modify DNA methylation status with consequent deregulation of PS1 and BACE and beta-amyloid production. Mol Cell Neurosci 28: 195-204.

16. Bassett SS, Avramopoulos D, Perry RT, Wiener H, Watson B, Jr., et al. (2006) Further evidence of a maternal parent-of-origin effect on chromosome 10 in late-onset Alzheimer's disease. Am J Med Genet B Neuropsychiatr Genet 141: 537-540.

17. Ehrenkrantz D, Silverman JM, Smith CJ, Birstein S, Marin D, et al. (1999) Genetic epidemiological study of maternal and paternal transmission of Alzheimer's disease. Am J Med Genet 88: 378-382.

18. Ogawa O, Zhu X, Lee HG, Raina A, Obrenovich ME, et al. (2003) Ectopic localization of phosphorylated histone H3 in Alzheimer's disease: a mitotic catastrophe? Acta Neuropathol (Berl) 105: 524-528.

19. Bolton SJ, Russelakis-Carneiro M, Betmouni S, Perry VH (1999) Non-nuclear histone H1 is upregulated in neurones and astrocytes in prion and Alzheimer's diseases but not in acute neurodegeneration. Neuropathol Appl Neurobiol 25: 425-432.

20. Casillas MA, Jr., Lopatina N, Andrews LG, Tollefsbol TO (2003) Transcriptional control of the DNA methyltransferases is altered in aging and neoplastically-transformed human fibroblasts. Mol Cell Biochem 252: 33-43.

21. Hemminki K, Lorenzo Bermejo J, Forsti A (2006) The balance between heritable and environmental aetiology of human disease. Nat Rev Genet 7: 958-965.
