# Supplementary material for: Age-Specific Epigenetic Drift in Late-Onset Alzheimer's Disease
Source: PLoS One. 2008 Jul 16;3(7):e2698. doi: 10.1371/journal.pone.0002698 (PMC2444024; doi:10.1371/journal.pone.0002698)
Supplement: Table S1 — (0.08 MB DOC) [file pone.0002698.s006.doc]

| Gene | **APH1B** |
| --- | --- |
| Primer: | F1: **AGGAAGAGAG**ATTATTTAAAAGGAGTTTAAATTTTTTT  R1: *CAGTAATACGACTCACTATAGGGAGA*AGGCTAACAATTCTCAAAAACATATATAATCCC |
| Amplified region | ATTATTTAAAAGGAGTTTAAATTTTTTTTTTTTTTTTTGAAGTTCGAATTTTATTTTTTAGATATTTTGATTTAATGGATCGGGGTGGGGGTGGGTATCGGTATGTTTTAAAGTATTTGGTTATAGGTGATTTTAATAGGCGGAAGTCGCGTATTATTATTTTAAACGTTAATGGTTTTTAATTTTTTGGGGATTATATATGTTTTTGAGAATT |
|  |  |
| Gene | **APOE promoter** |
| Primer: | F1: AGTTATGGAGATTTGAGGATATTGGGG  R1: TCAAAAAACCTCTATACCCCACCTCC  F2: **AGGAAGAGAG**GGATATTGGGGATATTTAGTAGGTGT  R2: *CAGTAATACGACTCACTATAGGGAGA*AGGCTAACCCTCTACCCTACTATACCTAAAACAA |
| Amplified region | GGACACTGGGGACACCCAGTAGGTGCTCGATAAATGATAGTGACAACTCGTGGAGTCCTGCTATGGCTTACATCCCAGTCCAGCTGCTCTCCCCACCCCACCTTCTAGCGGGTCGGGTCGTCTCTGCTGCCCAGCCCTTCCCAGGTCCAGTCCCCTGCTGCTTGCCTCACCCCCGCTCCTCCTCTCCCCAAGCCCGACCCCGAGTAGCTCTCCTGAGACTACCTGGAGGCCAGGGGTTCCCAGGGTCCCAGCTCTTTCTAGAGGCCCCTGAGCTCATCCCCGTGCCCCCGACTGCGCTTCTCACCGGCTCCTGGGGAAGGACGTCCTTCACCTCCGCTGGGGCTGAGTAGGACTCAAGGATCCCAGACTTGTCCAATTATAGGGCTCCCCCTGTCCCGCCCCCTCCCCCAGGGATAGGGCGGGCTGGGCCAGCCCCCAGTCACGAGGTGGGCTGTTCTCCCCCTGCCCCAGGCACAGCAGGGCAGAGG |
|  |  |
| Gene | **APOE 3’-CpG island** |
| Primer: | F1: GTAGAGAGAAAGATATATATAGATATAG  R1: CCTAAAACCCCTAATAAAACAAAAC  F2: **AGGAAGAGAG**GTTGAGAATTGTGTGGTTAGTATGT  R2: *CAGTAATACGACTCACTATAGGGAGA*AGGCTAACCCAAACTAAAAAAACAAACCCAAC |
| Amplified region | TGTAGGTTTTTAATTTTTTTATGGTTTCGTTTATTAGCGTTTTGCGGTCGAGAGGGCGGGAGGGTGTCGTATAGTGGGAGGCGGGGAGAAGGGTTGGGATGGGGCGGGCGAGATGGGGATGAGTTAGAGAGATTTAAGAGGGAGAGAAGGAACGGGGTAGAGGTCGAGAGAAGGAGATAGAGATAGATGTAGAGAGTAGATGTAGAGGG |
|  |  |
| Gene | **NCSTN** |
| Primer: | F1: GAAGGGATTTTTAGGTTTAGGTAAAGGGGGA  R1: CTAACCAAACCCAACCTCTCTCCCACC  F2: **AGGAAGAGAG**GGGAGGTTTTATTTGTTAGTAGGA  R2: *CAGTAATACGACTCACTATAGGGAGA*AGGCTAACCTAACATCCAACCTCCATATTC |
| Amplified region | GGGAGGCCTCACCTGCTAGTAGGACGCAGAAAGACAGAAGGCGAAGGAGACCCCGACTTCCCGGGTCAGCCCCAGAGCCACCCCCTGCCGTAGCCATCTTGCCTCTCTGCTGAGCGGAAGCCCCCGTTCGGCTCCTGTCTGTTAGCGGCCTCTCTAGGCTACCACTGACACCGTCTCTGTGGCCCGGAGCCTAAGAGACCGGAAGTTCGTGTTTCCAGGCGCTTCCGGAAACCGCGGGAGAGGGTCGCTGACGTGGAGGCGTCCGAAGGGCAGCAGGGTGTGTCGGGGCTCGGATTAAGACATCGGAGTCGGAGACCTGAGAGATGTTAACCAAATTCGAGACCAAGAGCGCGCGGGTCAAAGGTAGTGGAGAATAAAAGGGGGAACATGGAGGTTGGATGTCAG |
|  |  |
| Gene | **SIN3A** |
| Primer: | F1: **AGGAAGAGAG**TGGTAATGAGGAGGTGTTAGG  R1: *CAGTAATACGACTCACTATAGGGAGA*AGGCTAACACCATACTCCCACTCTCCTAACTA |
| Amplified region | TGGTAATGAGGAGGTGTTAGGGAAAGTTCGTTGTCGGGTTTGGTTGGGAGTTTCGGCGTTTAGATTTGTTTCGTTTTATGTTTTCGTGGGGAGGGAGGGGTATTCGTCGATTGGGAGTTGTGGAGGTGTTAGGATTTATTTTTGTGGTTGGGTTATGTTTTTTGTTTGGGGGTATAAGGGTTTTGTGTCGGAGTTGTTTTTTTCGTTTTGTTTTATGTAGCGTGTTTGGTATCGTGTAGTTAGGAGAGTGGGAGTATGGT |
|  |  |
| Gene | **TFAM** |
| Primer: | F1: TGGTAATATATAATTTTAGTAATTATAATTGGA  R1: CCTCTACTCAAATTAAACAAACTAAAAAC  F2: **AGGAAGAGAG**TAATAGATAGTTTTGTATTTAGGAT  R2: *CAGTAATACGACTCACTATAGGGAGA*AGGCTAACAAACCAAACTAAAAAACTACA |
| Amplified region | TAATAGATAGTTTTGTATTTAGGATTTCGAGGTTCGATCGGCGGTTGTTGCGCGTTTAGCGGAGCGTTTTAGTTTAAGTTAGGGTTGGAGTTCGGAGTTTAGAAATAGTAACGGGAGAGGCGGGGATTCGTAAATTTAAAAATGTAGATTTTAAGGTTTTATCGTAGAGTTTAATTTCGTGTGTTTATGTGGGGTTTATTAATTTTTATTTTAGCGAGTTTTTTAGAATTTAATAAGTTTTAATGTCGGTGGTTTTGAGATTATATTTAAAGTAATATAGTTTTAGATTGTTTATTTTATTATTTATTGTAGTTTTTTAGTTTGGTTT |
|  |  |
| Gene | **HTATIP** |
| Primer: | F1: TGTAGAGGGGGATTTTTGAGAGATAT  R1: CCTAATATACAAAATAAAACTAAAAATCTTCCC  F2: **AGGAAGAGAG**AGGGGTAAGATTGTTTTTGTGATT  R2: *CAGTAATACGACTCACTATAGGGAGA*AGGCTAACCTTCCCTAAATCCTCTTACCTA |
| Amplified region | AGGGGCAAGACTGCCCCTGTGACTCGGGGGAAAACGGAGTGTTACAAGCCTCAGGCCGAGCCCTAGGTGGAAACCCCATAACGTGCACAGCCAGTGGCGGTCTACTGAGTCCGTCACGTGACGCCCACCGGGTGCGAAGCTGGTCACGTGTATGCGGAGCGGGTCACGTGGCAATACCGTTTTTGGCTTTGTCGGAGGGAAGTCCTCGGGCGTCTCCTTGTGGCGGCGGGAGGTGTTGCCGGGAGTCAACTGGGCGCGAGAGGCTGGAGAAAGGAAGTGGCCTTAGAAGGGTTCGAACGCTCCCCCAAGTCTGTGGACTTCATTCCGTAGGCAAGAGGACTCAGGGAAG |
|  |  |
| Gene | **MTHFR** |
| Primer: | F1: GAGGGATAGAGTGAGAAAGGATTGGAGAAG  R1: TCTACAACCACTCCTAATCTCAATCCCAAAACT  F2: **AGGAAGAGAG**GTAGTATGATAAGTATAAAGTTTTGT  R2: *CAGTAATACGACTCACTATAGGGAGA*AGGCTAACCTCTATACTACTACTACAAATA |
| Amplified region | GCAGCATGATAAGCACAAAGTCCTGTGAGGAAGCTCATTCTGAAAACGCTTGTTTCATTCCAAACTCTTTTCAGATGGAAATAAAAGGAAACATGGGTGGGATTTACTGGAGCTGGCCTGGATTCTCCCTCAGATTCCAGGAGGGGTTATGAGAAAAGACCCCAGACTTAGGCACGTGAAGCAGGGTAGACGCTTCGAGAGCCCTGGCTGCGGTCCCCAGGCCCCACCCGCTGCCACCTGCGGGCCCAGATTGGCCCGGCCCCACCCCCGGCAACGCCTCTCTCAGTCCCTTAGCAACCGCCCCCTCCCCAGGCCGACTCCGCCGGCTTCTTACCAGCTCCTCGGGGGTGCGGGTCTCACGCTCACCGCAGCAGCAGCACCACCTGCAGCAGCAGCACAGAG |
|  |  |
| Gene | **APP** |
| Primer: | F1: GAATTTTGTTTTTAAGAAGAAGTAAATG  R1: TAAACAATAAAAAAAAAAATCTAAAACC  F2: **AGGAAGAGAG**AGGTTTTGTTGTTTTAATAAGTAAAG  R2: *CAGTAATACGACTCACTATAGGGAGA*AGGCTAACATACAAAATCAAAAAAAAATAAATCCT |
| Amplified region | AGGTTTTGCTGTCTCAACAAGCAAAGAAAATCCTATTTCCTTTAAGCTTCACTCGTTCTCATTCTCTTCCAGAAACGCCTGCCCCACCTCTCCAAACCGAGAGAAAAAACGAAATGCGGATAAAAACGCACCCTAGCAGCAGTCCTTTATACGACACCCCCGGGAGGCCTGCGGGGTCGGATGATTCAAGCTCACGGGGACGAGCAGGAGCGCTCTCGACTTTTCTAGAGCCTCAGCGTCCTAGGACTCACCTTTCCCTGATCCTGCAC |
|  |  |
| Gene | **PSEN1** |
| Primer: | F1: ATTAGTTGTTTAAGTAGATTTAGTTTA  R1: ctaaacccaatttatataaaaacttt  F2: **AGGAAGAGAG**TAGTTTAGGTTTTTTTTAGATTAGT  R2: *CAGTAATACGACTCACTATAGGGAGA*AGGCTAACAAAAACCAAATAAAACTCTAAATTC |
| Amplified region | TAGCTCAGGTTCCTTCCAGACCAGCCGCTGTTTTGTTTCCGATGTGAAACCGCGGACCCCGGAGCTGCCTGTCCCAGGCCCCGCCCGAGAACCCTCACCGTTGTCGTCATTTCCGGCTCTGGCGTCGTTGGCGGCCGGCCCACGGAGGAGGGGAGGAGCCAGGAGGAAGGGGGCGGGGCCTCCGGCCTGGAGAAACGATTGCGGGGAGAACCCAGAGCTCCACCTGGCCTTC |
|  |  |
| Gene | **BACE** |
| Primer: | F1: ATTGAGTTTTTTTAGATTTTTTAGATGT  R1: TAACTACTCAAACCACCATAATC  F2: **AGGAAGAGAG**TTTATGTTGAAAGAAAGATTGATAGA  R2: *CAGTAATACGACTCACTATAGGGAGA*AGGCTAACTCAAACCACCATAATCCAACT |
| Amplified region | TCCATGCTGAAAGAAAGACTGACAGACGGGAGGTGTGCCCCTCTCCATCCGTCTGGCCCTTCCCGCCAGGGCCTTGCAGGGCGGACTCCACCTCGGCAGAGGGCATCCCAGACCCCTCTCCAGCCCCGGAAGCCGGATTGCCTGCCATGGGAAGACTACACTTCCCAGCGATCCCAGGGAAAAGCGAAAACCTTTTGGCTTTGACAGCCGCCGCCACAAGTCTTTCCGCCTCCCCAGCCCGCCCGGGAGCTGCGAGCCGCGAGCTGGATTATGGTGGCCTGA |
|  |  |
| Gene | **DNMT1** |
| Primer: | F1: TAGTTTTTTGGTGGTTAGAAATTAGG  R1: TAAAATTCCTACCAAAACATAAACTTTCC  F2: **AGGAAGAGAG**GATTTTTAAATATAGTAAGTTTTGGGT  R2: *CAGTAATACGACTCACTATAGGGAGA*AGGCTAACTCCCAACTAACAAAATAAATACC |
| Amplified region | GATCCCCAAATACAGCAAGCTTTGGGTTCGTTTCCGGGGTCCCCTTCTTCAAGCAGCGGTGGGCCGGGCTCTTGACTCCAGCTTGGTCGCACAGGAAGTGGGCAGCCCGCGGCCAACGGACACCCCTCGGGCACCGGCACTTCGGCCTCCACTTCCGGTGTCCGGCCCGGTCCCCGGGGGCGCTTCTGTGGTGGGGGGTCCCTCAGTGCCTTTCCCCCAAAGCTGTGCATCTCGATGGCGGCCTCCAGAGACGGATGTCACCCGTCGGGGCCTGGGCTGGGCTGGCTCATGTTCGAGAGCCCAGCCTTCCGTGGGGTACTCATTCTGTCAGTTGGGA |

**Table S1: Primer sequences and interrogated genomic regions.** *Tag-sequences are marked in bold letters; the spacer sequences are underlined and the T7-promoter sequences are marked in cursive letters. CpG sites in the primer sequences were not included to avoid preferential amplification of methylated or unmethylated DNA. F2/R2 = primers for nested PCR reaction.
