# Supplementary material for: Age-Specific Epigenetic Drift in Late-Onset Alzheimer's Disease
Source: PLoS One. 2008 Jul 16;3(7):e2698. doi: 10.1371/journal.pone.0002698 (PMC2444024; doi:10.1371/journal.pone.0002698)
Supplement: Table S2 — (0.04 MB DOC) [file pone.0002698.s007.doc]

| **Parameter** | **Value** |
| --- | --- |
| Mean of distance (males) | 123 (sd = 32.6) |
| Mean of distance (females) | 137 (sd = 25.3) |
| The difference in the means | Not significant (p = 0.25) |
| The difference in the variances | Not significant (p = 0.21) |

**Table S2: Gender specific epigenetic distance (Euclidean distance**). Neither brain samples nor lymphocyte samples exhibit significant overall methylation difference between sexes.
